# Healthcare Resource Use and Expenditures among Metastatic Breast Cancer Patients Treated with HER2-Targeted Agents

**DOI:** 10.1155/2014/475171

**Published:** 2014-08-07

**Authors:** Nicole Meyer, Yanni Hao, Xue Song, Nianwen Shi, William Johnson, Jaqueline Willemann Rogerio, Denise A. Yardley

**Affiliations:** ^1^Truven Health Analytics, Cambridge, MA 02140, USA; ^2^Novartis Pharmaceuticals, East Hanover, NJ 07936-1080, USA; ^3^Sarah Cannon Research Institute, Nashville, TN 37203, USA; ^4^Tennessee Oncology, PLLC, Nashville, TN 37203, USA

## Abstract

*Objective*. To compare healthcare utilization (HCU) and costs of women newly diagnosed with metastatic breast cancer (mBC) by receipt of HER2-targeted agents (H2T) and among H2T subgroups. *Methods*. Adult women newly diagnosed with mBC (index date) during 2008–2012 were followed until enrollment end or inpatient death. Study cohorts were antineoplastic ± H2Ts, and no treatment; and subgroups of H2T patients stratified by receipt of hormonal therapy (HT+/HT−), by *de novo* versus recurrent disease status, and by age group. All-cause (ALL) and breast cancer related (BCR) HCU and costs (in 2012 dollars) were estimated using a generalized linear model. *Results*. Of 18,059 women, 14.6% were H2T users 71.1% nonusers, and 14.3% untreated. No treatment patients had the highest ALL and BCR inpatient HCU, and ALL emergency room HCU. H2Ts users had the highest ALL and BCR office visits, lab and diagnostic radiology, radiation treatments, other outpatient services, and prescription antineoplastics. Adjusted ALL and BCR costs were the highest for H2T users and, in H2T subgroups, higher for HT—versus HT+ and *de novo* versus recurrent, and declined with older age. *Conclusions*. Receipt of H2Ts was associated with greater levels of ALL and BCR HCU and costs. H2T subgroups of HT−, *de novo*, and younger age had higher HCU and costs, possibly indicating more aggressive treatments.

## 1. Introduction

The American Cancer Society estimates that more than 230,000 women will be diagnosed with and about 40,000 women will die from breast cancer in 2014 [1]. Approximately 6–10% of breast cancer patients are initially diagnosed with metastatic (stage IV) disease [2], while an additional 20–40% of patients with early stage breast cancer eventually develop stage IV metastases [3]. Long-term survival in patients with metastatic breast cancer (mBC) is influenced by a variety of factors including human epidermal growth factor receptor 2 (HER2) status, hormone receptor (ER) status, age at diagnosis, and sites of metastases [4]. In particular, approximately 1 in 5 breast cancer tumors are HER2-positive, which is associated with faster tumor growth and higher likelihood of recurrent cancer [2]. In 2006, the U.S. Food and Drug Administration (FDA) approved the first targeted therapy, trastuzumab, for all HER2-positive breast cancers. Three newer HER2-targeted agents, lapatinib, pertuzumab, and ado-trastuzumab emtansine, entered in the US market between 2007 and 2013 [5]. Together, they have significantly improved the prognosis for HER2-positive patients with recurrent and metastatic breast cancer [6].

Breast cancer remains one of the most costly cancers in the United States [7], with an estimated $16.5 billion spent on breast cancer treatment in the US in 2010 [8]. Several recent studies have examined the cost of treating patients with mBC [9–12]. Based on 2005–2009 health insurance claims, Ray et al. reported the average per patient per month (PPPM) cost of $13,147, $11,610, and $10,219 during the first 6 months, 7–12 months, and 12–24 months following mBC diagnosis [11]. Using 2003–2009 medical claims, Montero et al. estimated an average PPPM of $5,303, $10,083, $8,847, $13,261, and $13,926 for patients with endocrine therapy only, HER2-targeted therapy only, HER2-targeted therapy + endocrine therapy, cytotoxic chemotherapy, and no-systemic therapy over a mean of 2.2-year follow-up, respectively [12]. However, to our knowledge, there is no published healthcare resource analysis on the following clinically relevant subgroups of mBC patients receiving HER2-targeted therapies: hormonal therapy status,* de novo* versus recurrent metastatic disease, and age. To fill this knowledge gap, we conducted an analysis to compare the healthcare utilization and expenditures among women with mBC stratified by receipt of HER2-targeted agents and further compared these outcomes among patients receiving HER2-targeted agents by receipt of hormonal therapy,* de novo* versus recurrent disease, and age group.

## 2. Methods

### 2.1. Data Source

Data on healthcare utilization and costs from 2007 through 2012 were extracted from the MarketScan Commercial and Medicare Supplemental Databases. The Commercial Database contains the integrated patient-level pharmacy and medical (inpatient and outpatient) claims of employees and their dependents, covered under a variety of fee-for-service and capitated health plans. Medical claims are linked to outpatient prescription drug claims and person-level enrollment data through the use of unique enrollee identifiers. The Medicare Database profiles the healthcare experience of individuals with Medicare supplemental insurance paid for by employers. Both databases provide detailed cost, utilization, and outcomes data for healthcare services performed in both inpatient and outpatient settings. All study data were fully compliant with United States patient confidentiality requirements, including the Health Insurance Portability and Accountability Act of 1996. Only statistically deidentified patient records were used, thereby exempting the study from Institutional Review Board approval.

### 2.2. Study Design

This longitudinal, retrospective, observational study included adult female patients with incident metastatic (stage IV) breast cancer (mBC) between January 1, 2008 and December 31, 2011. The date of the first medical claim for metastasis was defined as the index date. Eligible patients were followed from initial mBC diagnosis to inpatient death, disenrollment from MarketScan, or the end of study at December 31, 2012, whichever occurred first.

The initial sample was divided into three mutually exclusive cohorts based on the presence or absence of claims for HER2-targeted agents (trastuzumab, lapatinib, or pertuzumab) during the entire study period (i.e., preperiod through follow-up). The HER2-targeted agent patients had a prescription or medical claim for trastuzumab, lapatinib, or pertuzumab at any point in the study period. No HER2-targeted agent patients had a prescription or medical claim for chemotherapy, hormone therapy, or non-HER2 biologic therapy but no evidence of HER2-targeted agents in the study period. No treatment patients had no medical or pharmacy claims for any chemotherapy, hormone therapy, HER2-targeted agent, or non-HER2 biologic therapy during the study period.

Patients receiving HER2-targeted agents were further stratified by hormonal therapy (HT) status into HT− (patients without medical or pharmacy claims for any HT) or HT+ (those with HT related claims). Status of metastasis as* de novo*—defined by 90 or fewer days between initial breast cancer diagnosis and index—or recurrent—defined by more than 90 days between initial breast cancer diagnosis and index—as well as age group (age 18–44, age 45–64, and age 65+) at index was used as additional levels of stratification.

### 2.3. Patient Selection

This study initially identified female patients 18 years or older with at least 1 inpatient or 2 nondiagnostic (e.g., no laboratory or diagnostic radiology) outpatient claims at least 30 days apart with a primary or secondary diagnosis code of breast cancer (*International Classification of Diseases, *9th* Revision, Clinical Modification* diagnosis code 174.xx) between January 1, 2008 and December 31, 2011. Patients were further required to have a diagnosis for stage IV breast cancer recorded on a nondiagnostic claim within 60 days prior to or subsequent to any breast cancer diagnosis. Cancer stage as defined by the American Joint Committee on Cancer (AJCC) (American Cancer Society) is not available in administrative claims data; therefore, ICD-9-CM diagnosis codes for secondary malignant neoplasms (ICD-9-CM diagnosis 196.1-196.2, 196.5-196.6, 196.8-196.9, 197.0–197.8, 198.0-198.1, 198.3–198.8, 198.82, 198.89, or 199.0-199.1) were used to proxy the corresponding AJCC listed location of metastases. Patients were excluded if they did not have at least 12 months of continuous medical and prescription coverage prior to the index date (preperiod), had a diagnosis for mBC in the preperiod, had a diagnosis for primary cancer other than breast cancer in the preperiod, or were diagnosed with AIDS/HIV or pregnancy at any point during the study period.

### 2.4. Study Variables

#### 2.4.1. Patient Demographic and Clinical Characteristics

Patient demographic variables such as age, gender, geographical location (US census division), population density, primary payer, and plan type were measured on the index date. Clinical characteristics were measured in the 12-month preperiod and included sites of metastases at index date, diagnosis of earlier stage breast cancer, and, where found, breast cancer-related surgical treatment (lumpectomy or mastectomy), radiation therapy, and hormonal, chemotherapy, HER2-targeted agent, or non-HER2 biologic/HER2-targeted agent treatments. The Deyo and Romano adaptation of the Charlson Comorbidity Index (CCI) [13] and the National Cancer Institute modification of the CCI (NCCI) were used to estimate the burden of illness [14]. The NCCI is an aggregate measure of cancer-specific comorbidity and excludes all cancer-related diagnoses. Comorbid conditions, including anemia, anxiety/depression, cardiac arrhythmia, cerebrovascular disease, congestive heart failure, coronary artery disease, chronic obstructive pulmonary disorder, diabetes, and hypertension were also recorded.

#### 2.4.2. Healthcare Utilization and Costs

All-cause and breast cancer-related healthcare utilization and costs were assessed in the postindex period for each of the study cohorts. Breast cancer-related costs and utilization were identified by inpatient or outpatient claims with a primary diagnosis for breast cancer (ICD-9-CM diagnosis 174.xx), and medical and prescription drug claims for chemotherapy, hormonal, HER2-targeted, or non-HER2 biologic agents.

Due to the variable length follow-up, all cost/utilization outcomes were standardized and reported at the mean per patient per month (PPPM) level. Specific utilization measures included inpatient admissions, total days of hospitalization, outpatient utilization (emergency department (ED) visits, physician office visits, radiation treatment, diagnostic radiology, laboratory services, and other outpatient care), and pharmacy prescriptions.

Healthcare expenditure data were collected for inpatient services, outpatient services, outpatient pharmacy, and total healthcare. Healthcare costs were based on paid amounts of adjudicated claims and included insurer payments (including coordination of benefits (COB)) as well as patient cost-sharing in the form of copayments, deductibles, and coinsurance. Costs for services provided under capitated arrangements were estimated with payment proxies based on paid claims at the procedure level using the MarketScan Commercial and Medicare Supplemental Databases. All dollar estimates were inflated to 2012 US dollars using the Medical Care Component of the Consumer Price Index (CPI).

### 2.5. Statistical Analysis

Descriptive analyses were performed to compare healthcare utilization and expenditures between study cohorts. Categorical variables were summarized by frequency and percentage. Continuous variables were reported by mean and standard deviation (SD). Statistical comparisons were evaluated using chi-square or exact tests for categorical measures and either ANOVA or parametric tests for continuous measures depending on the distributional properties of the specific measure evaluated.

Multivariate analyses were also conducted to estimate total all-cause and breast cancer-related costs. Mean costs per patient per month were modeled with a generalized linear model assuming an underlying gamma distribution and a log link relating the mean costs to a set of covariates or predictors. Covariates included age group, geographic location, urban or rural location, type of insurance, hormonal therapy status,* de novo* status, Deyo Charlson Comorbidity Index, preindex breast cancer surgery, preindex radiation therapy, and preindex use of hormonal, non-HER2 biologics/HER2-targeted agents, or chemotherapy agents. Incremental costs (i.e., difference in means) were calculated via least squares means inversely transformed back onto the original dollar scale. Mean expenditures for each group of interest and the incremental expenditure difference between the two group means were summarized with means, standard errors (SE), and 95% confidence intervals (CI).

## 3. Results

### 3.1. Study Population

Of the 518,630 women diagnosed with breast cancer during January 1, 2008, through December 31, 2011, 59,014 (11.4%) had a stage IV metastatic diagnosis. After screening for age (*n* = 59,007), continuous enrollment (*n* = 26,456), and exclusionary diagnoses (*n* = 18,059), a total of 18,059 women were eligible for study. Of the eligible population, 2,629 (14.6%) were treated with a HER2-targeted agent, 12,840 (71.1%) were treated with antineoplastics other than HER2-targeted agents, and 2,590 (14.3%) were untreated. Among the 2,629 patients using HER2-targeted agents, 1,357 (51.6%) were HT+ and 975 (37.1%) were* de novo*. Four hundred and four patients (15.4%) were aged 18–44, 1,801 (68.5%) were aged 45–64, and 424 (16.1%) were 65 and older at index.

### 3.2. Demographic and Clinical Characteristics

#### 3.2.1. Patients Receiving HER2-Targeted Agents, No HER2-Targeted Agents, and No Treatment

Patients receiving HER2-targeted agents were, on average, 56 years old, which was 4 and 6 years younger than those in the no HER2-targeted agents and no treatment cohorts, respectively (*P* < 0.001). The majority of the patients were covered by a preferred provider organization (PPO)/exclusive provider organization (EPO) plan in all the three cohorts with subtle differences in the other insurance plans (*P* < 0.001) (Table 1).

The proportion of patients with evidence of breast cancer in preindex was similar in the HER2-targeted (72.7%) and no HER2-targeted cohorts (71.2%), and higher than the no treatment cohort (66.7%) (*P* < 0.001). Among the three treatment cohorts, the HER2-targeted cohort had the highest preperiod treatment rate with breast cancer-related surgery (26.0%, *P* < 0.001), radiation therapy (17.9%, *P* < 0.001), and chemotherapy (44.5%, *P* < 0.001). The rate of preperiod hormonal therapy was lower for the HER2-targeted group than no HER2-targeted agent patients (both *P* < 0.001). Nearly 2 in 3 patients in the HER2-targeted cohort were treated with HER2-targeted agents prior to their mBC diagnosis.

The HER2-targeted cohort had a slightly higher CCI score than the other two cohorts (*P* < 0.001). At index, HER2-targeted agent users had more sites of metastasis (*P* < 0.001) and were more likely to have brain (*P* < 0.001) and liver (*P* < 0.001) metastases, whereas metastases to bone were more common in no HER2-targeted agent patients. Lung metastases were more common in the no treatment group (*P* < 0.001).

#### 3.2.2. HER2-Targeted Agent Subgroups

Baseline patient characteristics and treatment patterns among the HER2-targeted agent subgroups are presented in Table 2. Patient demographic profiles were similar across HER2-targeted agent subgroups by HT+ status and* de novo* versus recurrent disease.

Baseline comorbid conditions as measured by CCI were lower for HT+ versus HT− (*P* = 0.002), higher for* de novo* versus recurrent (*P* < 0.001), and were similar across age groups. HT+ patients had fewer metastases sites at index (*P* = 0.004) and were less likely to have liver (*P* < 0.001), lung (*P* < 0.001), and brain (*P* < 0.001) but more likely to have bone (*P* < 0.001) metastases than HT− patients. During the preindex period, HT+ patients were less likely to receive chemotherapy (*P* < 0.001) and HER2-targeted agents (*P* < 0.001) compared to HT− patients. Although* de novo* patients had more index metastases sites (*P* < 0.001), they were less likely to have brain (*P* < 0.001) and lung (*P* < 0.001) metastases than recurrent patients. Among the three age groups, use of hormone (*P* = 0.005), chemotherapy (*P* = 0.037), and HER2-targeted agents (*P* = 0.004) was the highest for ages 18–44 and declined with age (except for hormone therapy).

### 3.3. Healthcare Utilization

Table 3 summarizes the unadjusted healthcare utilization of study patients by receipt of HER2-targeted agents. There were significant differences in a number of utilization measures among the three treatment cohorts. Specifically, compared to the no treatment cohort, patients in the no HER2-targeted agent and HER2-targeted agent cohorts had significantly lower all-cause and breast cancer-related PPPM inpatient admissions and inpatient days and all-cause ED visits (all *P* < 0.001). However, the HER2-targeted agents cohort had the highest all-cause and breast cancer-related outpatient office visits, laboratory, diagnostic radiology, radiation treatments, and other outpatient care (all *P* < 0.001). Monthly utilization for all-cause prescription fills was the highest in those receiving no HER2-targeted agents (*P* < 0.001). The monthly mean number of claims for office-administered antineoplastic agents was higher for the HER2-targeted cohort compared to no HER2-targeted agents (0.04 versus 0.01, *P* < 0.001).

Among HER2-targeted agent users, HT+ patients had significantly lower monthly mean number of all-cause inpatient admissions (0.09 versus 0.20), inpatient days (0.50 versus 1.00), ED visits (0.08 versus 0.17), outpatient office visits (1.85 versus 2.03), and number of claims for other outpatient care (3.82 versus 4.53) than the HT− patients (all *P* < 0.0001). A similar trend was seen in breast cancer-related utilization for all service types except breast cancer-related office and outpatient antineoplastics (Table 4).

In comparison to the* de novo* cohort, patients in the recurrent cohort had significantly higher all-cause healthcare utilization for inpatient admissions (0.16 versus 0.11), inpatient days (0.81 versus 0.62), and ED visits (0.14 versus 0.09), but lower utilization for outpatient services including office visits (1.87 versus 2.05), diagnostic radiology (2.06 versus 2.35), laboratory services (5.01 versus 5.71), and other outpatient care (4.07 versus 4.33). However, with the exception of ED visits and number of prescriptions for outpatient antineoplastic agents, recurrent patients had significantly lower utilization in both breast cancer-related inpatient and outpatient services than the* de novo* patients (Table 4).

All-cause PPPM utilization for inpatient days, ED visits, other outpatient services, and prescription fills increased with older age for HER2-targeted agent users. While the PPPM number of outpatient visits, radiation treatments, diagnostic radiology, and laboratory services declined with age, only laboratory services showed statistical significance (*P* < 0.001). With the exception of inpatient days, ED visits and office-administered antineoplastics, all other PPPM breast cancer-related utilization declined with older age (Table 4).

### 3.4. Healthcare Expenditures

Patients with HER2-targeted agents had the highest unadjusted total PPPM healthcare expenditures for all-cause and breast cancer-related services among the three treatment cohorts, amounting to $14,105 PPPM for all-cause expenditure and $8,585 for breast cancer-related services (all *P* < 0.001) (Table 5).

Higher costs in the HER2-targeted cohort were primarily driven by other outpatient care ($7,083) which captured the expensive anticancer treatments including HER2-targeted agents as well as the cost of other medications administered in an outpatient setting including those with indications for side effects of antineoplastics (e.g., antiemetics or granulocyte colony-stimulating factors). The no treatment cohort incurred higher expenditures for all-cause ($6,683 no treatment, $3,416 no HER2-targeted agents, and $3,327 HER2-targeted agents; *P* < 0.001) and breast cancer-related inpatient admissions ($1,525 no treatment, $271 no HER2-targeted agents, and $338 HER2-targeted agents; *P* < 0.001) than those in the other cohorts.

Other outpatient care, which included systemic and anticancer treatments administered in the outpatient setting, accounted for the largest component of total all-cause and breast cancer-related expenditures in HER2-targeted and no HER2-targeted cohorts (48.6% of total breast cancer-related costs for HER2-targeted agent users and 48.1% for no HER2-targeted agent users), while inpatient costs were the largest cost component for no treatment patients. Inpatient costs for the HER2-targeted and no HER2-targeted agent cohorts represented 24% and 35% of total all-cause costs, respectively. In contrast, all-cause inpatient costs accounted for nearly 60% of total PPPM costs in the no treatment cohort. Similarly, while inpatient costs in the HER2-targeted and no HER2-targeted agent cohorts were 4–6% of total breast cancer-related costs, they represented 37% of total breast cancer-related costs in the no treatment cohort.

Among HER2-targeted agent users, total unadjusted all-cause and breast cancer-related expenditures were significantly lower for HT+ (all-cause: $12,391 versus $15,934, *P* < 0.001; breast cancer-related: $8,092 versus $9,103, *P* < 0.01) and recurrent patients (all-cause: $13,446 versus $15,223, *P* < 0.01; breast cancer-related: $7,510 versus $10,409, *P* < 0.001) compared to their respective counterparts (Table 6). Healthcare costs were similar for the 18–44 and 45–64 year age groups, whereas the age 65+ cohort had the lowest costs.

### 3.5. Multivariate Adjusted Healthcare Expenditures

#### 3.5.1. Patients Receiving HER2-Targeted Agents, No HER2-Targeted Agents, and No Treatment

The adjusted PPPM all-cause healthcare expenditures among patients receiving HER2-targeted agents were $11,107 (95% CI: $10,376–$11,838), $2,649 (95% CI: $1,892–$3,405) higher than no HER2-targeted agent patients and $2,824 (95% CI: $2,009–$3,638) higher than no treatment patients (Table 7). Similarly, the HER2-targeted cohort had incremental breast cancer-specific costs of $2,307 (95% CI: $1,817–$2,796) and $3,357 (95% CI: $2,855–$3,859) compared to the no HER2-targeted cohort and the no treatment cohort, respectively. This suggests the cost differential was largely driven by breast cancer-specific services. Younger age (age 18–44),* de novo* status, preindex chemotherapy, and higher preindex CCI were associated with increased total all-cause and breast cancer-related expenditures (*P* < 0.05). Older age (age 65+), comprehensive and health maintenance organizations (HMO) insurance plans, preindex breast cancer surgery, preindex hormone therapy, and residence in the Northeast, North Central, and Western regions of the United States (relative to South) were negatively associated with all-cause and breast cancer-specific costs. Preindex radiation therapy was associated with higher all-cause expenditures (*P* < 0.001). Point-of-service (POS) insurance plan was negatively associated with breast cancer-related (*P* < 0.05) but was insignificant for all-cause expenditures.

#### 3.5.2. HER2-Targeted Agent Subgroups

Among patients receiving HER2-targeted agents, HT+ patients had adjusted PPPM all-cause costs of $10,359 and breast cancer-specific costs of $6,869, which were $2,565 lower (95% CI: −$3,397 to −$1,733) and $888 lower (95% CI: −$1,472 to −$305), respectively, than the HT− patients.

*De novo* status resulted in a statistically significant increase in the total all-cause healthcare costs from $11,184 (95% CI: $10,689–$11,679) to $12,139 (95% CI: $11,438–$12,840), an increase of $955 (CI: $97–$1,813) and in breast cancer-specific expenditures from $6,791 (95% CI: $6,450–$7,131) to $8,200 (95% CI: $7,674–$8,726), an increase of $1,409 (95% CI: $782–$2,036), respectively (*P* < 0.05).

Consistent with descriptive data, HER2-targeted patients in age groups of 18–44 and 45–64 had similar adjusted all-cause healthcare costs, but the age 65+ cohort was $4,531 lower (95% CI: −$5,323 to −$3,739) when comparing to age group 45–64. The same pattern was observed in breast cancer-specific costs, as the 65+ cohort was estimated to have costs $2,995 lower (95% CI: −$3,548 to −$2,443) than the age 45–64 cohort.

Preindex hormone therapy,* de novo* status, and preindex CCI were associated with significantly higher total all-cause expenditures (*P* < 0.05). Similarly, younger age,* de novo* status, and preindex CCI were associated with significantly higher breast cancer-related expenditures (*P* < 0.05). Having comprehensive or HMO health plan, evidence of preindex breast cancer surgery and preindex use of non-HER2-biologic or HER2-targeted agents were related to lower all-cause and breast cancer-specific expenditures (*P* < 0.05). In addition, enrollment in a POS health plan was associated with lower breast cancer-specific expenditures (*P* < 0.05).

## 4. Discussion

Breast cancer care poses a significant financial burden on the US healthcare system. The introduction of more effective but costly targeted therapies in the treatment of mBC has contributed to the rise of healthcare resource use for mBC patients. While previous studies have estimated the costs of treating mBC patients, only one study has calculated costs for patients receiving HER2-targeted therapies [12]. This study examines the healthcare utilization and costs of treating mBC patients receiving HER2-targeted agents, stratified by subgroups in a large population, by capturing the full spectrum of real-world mBC patient experiences. In addition, the data are derived from a geographically diverse range of 100 health plans from a broad spectrum of plan types with US coverage from 2008 to 2012, making these latest estimates representative of the US managed health care population. The mBC population in this study includes adult female patients, insured by commercial or Medicare plus Medicare supplemental medical and prescription plans, receiving mBC chemotherapy, hormone therapy, and HER2-targeted or non-HER2-biologic agents treatment alone or in combination across multiple years.

This study found an average mean monthly all-cause expenditure of $11,107 per patient among users of HER2-targeted agents, $8,458 for patients with no HER2-targeted agents, and $8,284 for patients receiving no treatment. Differences in total unadjusted healthcare expenditures between patients with and without HER2-targeted agents (~$4,277) were primarily driven by differences in other outpatient care (~$3,422), which included outpatient systemic anticancer treatments and other medications (e.g., those used to treat side effects of antineoplastics) administered in the outpatient setting. Similarly, differences in expenditures directly related to treating breast cancer were largely attributable to other outpatient care (including medications indicated to treat side effects of antineoplastics) and the cost of antineoplastics. Other outpatient care costs accounted for the largest component of all-cause and breast cancer-specific expenditures for patients with and without HER2-targeted agents: all-cause, 50% and 37%; breast cancer-related, 49% and 48%, respectively. Moreover, results of this study demonstrated that among HER2-targeted users, expenditures were lower among recipients of hormonal therapies (as a proxy for endocrine-receptor positive status) compared to those without hormonal therapies, higher for* de novo* than for recurrent patients, and was the lowest in patients aged 65 and older. Specifically, the average total all-cause costs were approximately $2,565 (25%) lower for HT+ patients than HT− patients, and $955 (8%) higher among* de novo* patients compared to recurrent patients. Average total expenditures for HER2-targeted agent patients aged 18–44 were $13,650 per month, slightly lower for patients aged 45–64 ($13,101), and significantly lower for patients aged 65+ ($8,570).

The most comparable data in the literature is presented by Montero et al. who estimated the treatment costs among mBC patients by treatment modality [12]. In their study, Montero and colleagues estimated the total overall healthcare expenditures of women with mBC, aged 18–64 and insured with managed care to be $9,788 PPPM across all patients, and $10,083 PPPM among patients taking HER2 therapy. These results were similar to our finding of $11,107 PPPM treatment costs among HER2-targeted agent users. Montero also estimated total PPPM costs among patients treated with HER2 and endocrine therapy to be $8,847 PPPM, which are somewhat lower than our estimates of $10,039 for HT+ patients receiving HER2-targeted therapies. Consistent with our study, several recent studies have also shown that outpatient expenditures are the largest component of total healthcare expenditures among metastatic breast cancer patients [10–12]. Montero et al. found outpatient care was the largest component of total healthcare costs, primarily driven by the costs of anticancer treatments [12]. Patients in HER2-targeted agent cohorts had anticancer treatments that were 48–50% of total healthcare costs. Likewise, in a study of postmenopausal women with metastatic breast cancer, Lage et al. reported unadjusted outpatient costs to be $57,820 per year, about 66% of the total costs [10].

This study has some limitations that are general to retrospective studies using administrative claims. Diagnostic and procedural information in administrative claims are recorded for the purposes of reimbursement; thus the identification of breast cancer patients and clinical outcomes are subject to incomplete and miscoded claims. In addition, because metastatic disease staging as defined by AJCC is not available in claims data, the identification of mBC relied on the accuracy of claims coding for secondary malignant neoplasms. Therefore, the study may have excluded some breast cancer patients with mBC. Similarly, claims do not contain information on the pathology of breast cancer tumors; HER2 and ER tumor-receptor status were inferred using the specific antineoplastics used by patients. Thus, untreated HER2+ or HER2+/ER+ may have been excluded from HER2-targeted agent cohorts. Finally, the MarketScan databases are comprised of the healthcare experiences of individuals with commercial health coverage or private Medicare supplemental coverage; thus results may not be generalizable to uninsured or Medicaid-insured mBC patients.

## 5. Conclusions

To our knowledge, this is the first study to provide a comprehensive cost comparison among US mBC patients by receipt of HER2-targeted agents and among clinically relevant subgroups of patients using HER2-targeted agents. Our study found total expenditures among mBC patients treated with HER2-targeted agents averaging $11,107 per patient per month. Furthermore, study findings suggest that there are significant differences in the use and expenditures for health care among subgroups of mBC patients receiving HER2-targeted agents; specifically, the receipt of HER2-targeted agents, HT− status,* de novo* disease, and younger age are significant drivers of increased expenditures. This retrospective analysis highlights the significant economic burden that HER2+ mBC represents to health plans and self-insured employers. As new and expensive targeted therapies become available in the treatment of HER2+ mBC, understanding the economic burden of HER2+ mBC will be important in planning for future healthcare costs and setting priorities for allocating healthcare resources.

## Figures and Tables

**Table 1:** Demographic characteristics and clinical history by receipt of HER2-targeted agents.

	No treatment	No HER2-targeted agents	HER2-targeted agents
All patients—*N*	*N* = 2,590	*N* = 12,840	*N* = 2,629

Age, mean (SD)	62.3 (13.6)	60.1 (12.7)	55.6 (11.2)^a^
Urban	82.8%	84.4%	83.6%
Payer			a
Commercial	64.0%	68.4%	83.5%
Medicare	36.0%	31.6%	16.6%
Insurance plan type^d^			a
Comprehensive	16.6%	15.5%	9.6%
EPO or PPO	52.6%	51.3%	57.1%
POS	6.6%	7.7%	9.1%
HMO	16.1%	16.8%	15.3%
CDHP or HDHP	3.2%	3.6%	4.0%
Unknown	4.8%	5.2%	5.1%
Earlier stage breast cancer diagnosis	66.7%	71.2%	72.7%^a^
Surgery for breast cancer^e^	15.6%	22.0%	26.0%^a^
Adjuvant/neoadjuvant treatment^f^	n/a	66.8%	77.8%^a^
Radiation therapy^e^	10.7%	15.4%	17.9%^a^
Any antineoplastic treatment^e^	n/a	75.4%	79.4%^a^
Hormone therapy	n/a	55.4%	33.9%^a^
Chemotherapy	n/a	27.8%	44.5%^a^
Non-HER2 biologic/HER2-targeted agent	n/a	4.9%	63.2%^a^
HER2-targeted agents	n/a	n/a	62.4%
Charlson Comorbidity Index (CCI), mean (SD)	3.41 (2.37)	3.71 (2.63)	3.87 (2.75)^a^
NCCI, mean (SD)	1.66 (1.05)	1.54 (0.97)	1.45 (0.89)^a^
Comorbidities			
Anemia	11.9%	12.5%	14.0%^c^
Anxiety/depression	7.4%	7.5%	8.7%
Cardiac arrhythmia	10.4%	9.5%	6.5%^a^
Cerebrovascular disease	6.0%	4.3%	2.3%^a^
Congestive heart failure	4.3%	3.2%	3.1%^c^
Coronary artery disease	7.7%	6.9%	5.2%^b^
Chronic obstructive pulmonary disorder	6.0%	5.3%	3.2%^a^
Diabetes	14.8%	14.6%	11.4%^a^
Hypertension	38.9%	37.3%	29.2%^a^
Number of metastasis sites at index, mean (SD)	1.48 (0.84)	1.54 (0.82)	1.62 (0.88)^a^
Site of metastasis at index			
Liver	12.4%	12.1%	17.2%^a^
Lung	14.9%	12.1%	13.5%^b^
Bone	29.5%	42.2%	36.2%^a^
Brain	11.9%	8.1%	14.0%^a^

*N* = number of patients meeting study selection criteria.

^a^ *P* value compared with no treatment and no HER2-targeted agents <0.0001.

^b^ *P* value compared with no treatment and no HER2-targeted agents <0.01.

^c^ *P* value compared with no treatment and no HER2-targeted agents <0.05.

^d^EPO: exclusive provider organizations, PPO: preferred provider organization plans, POS: point-of-service, HMO: health maintenance organization, CDHP: consumer-driven health plan, and HDHP: high deductible health plan.

^e^The denominator for the percentages is the number of patients with a diagnosis of an earlier stage breast cancer. ^f^Percent of patients with surgery in the preindex period.

**Table 2:** Demographic characteristics and clinical history among patients with HER2-targeted agents.

	Patients with HER2-targeted agents						
	HT−	HT+	*De novo *	Recurrent	Age 18–44	Age 45–64	Age 65+
All patients—*N*	*N* = 1272	*N* = 1357	*N* = 975	*N* = 1654	*N* = 404	*N* = 1801	*N* = 424

Age, mean (SD)	55.7 (10.8)	55.4 (11.6)	55.2 (11.4)	55.7 (11.2)	38.6 (4.3)	55.1 (5.5)	73.6 (6.3)^a^
Urban	83.3%	83.9%	82.9%	84.1%	87.6%	82.8%	83.3%
Payer							
Commercial	84.3%	82.7%	84.3%	83.0%	100%	99.3%	0.5%
Medicare	15.7%	17.3%	15.7%	17.0%	0%	0.7%	99.5%
Insurance plan type^d^							a
Comprehensive	10.3%	8.8%	9.0%	9.9%	1.7%	4.1%	40.1%
EPO or PPO	56.5%	57.6%	58.6%	56.2%	56.9%	60.6%	42.0%
POS	8.5%	9.6%	8.6%	9.3%	11.4%	10.0%	2.8%
HMO	15.2%	15.3%	13.9%	16.1%	19.3%	14.7%	13.7%
CDHP or HDHP	4.3%	3.8%	4.3%	3.9%	6.2%	4.5%	0.0%
Unknown	5.2%	4.9%	5.6%	4.7%	4.5%	6.1%	1.4%
Earlier stage breast cancer diagnosis	73.1%	72.3%	26.5%	99.9%^a^	70.1%	72.6%	75.5%
Surgery for breast cancer^e^	27.7%	24.3%	41.9%	23.5%^a^	33.9%	25.5%	20.9%^b^
Adjuvant/neoadjuvant treatment^f^	75.2%	78.6%	39.8%	87.1%^a^	77.1%	77.8%	71.6%
Radiation therapy^e^	18.6%	17.2%	6.6%	19.7%^a^	24.4%	17.6%	13.4%^c^
Any antineoplastic treatment^e^	70.9%	87.5%^a^	49.2%	84.1%^a^	85.9%	78.8%	75.9%^b^
Hormone therapy	0.0%	66.1%^a^	14.0%	37.0%^a^	39.6%	31.5%	38.8%^b^
Chemotherapy	50.0%	39.3%^a^	36.0%	45.9%^b^	47.0%	45.6%	38.1%^c^
Non-HER2 biologic/HER2-targeted agents	67.4%	59.2%^a^	34.1%	67.8%^a^	67.1%	64.2%	55.6%^b^
HER2-targeted agents	66.7%	58.4%^b^	33.7%	66.9%^a^	66.8%	63.4%	54.7%^b^
Charlson Comorbidity Index (CCI), mean (SD)	4.05 (2.84)	3.71 (2.65)^b^	4.38 (2.99)	3.62 (2.58)^a^	3.88 (2.75)	3.83 (2.75)	4.03 (2.74)
NCCI mean (SD)	1.47 (0.93)	1.44 (0.85)	1.35 (0.76)	1.50 (0.76)^a^	1.28 (0.66)	1.37 (0.77)	1.69 (1.12)^a^
Comorbidities							
Anemia	14.4%	13.6%	7.4%	17.9%	11.9%	13.9%	16.5%
Anxiety/depression	9.4%	8.0%	8.5%	8.8%	12.9%	8.3%	6.4%^b^
Cardiac arrhythmia	6.4%	6.6%	7.2%	6.2%	3.5%	5.1%	15.8%^a^
Cerebrovascular disease	2.5%	2.1%	2.5%	2.2%	0.5%	1.8%	6.1%^a^
Congestive heart failure	3.8%	2.4%^c^	1.3%	4.1%^a^	0.7%	2.9%	5.9%^a^
Coronary artery disease	4.3%	6.0%	4.7%	5.4%	1.0%	4.3%	12.7%^a^
Chronic obstructive pulmonary disorder	3.9%	2.5%^c^	3.4%	3.0%	0.5%	2.9%	6.8%^a^
Diabetes	12.4%	10.5%	10.8%	11.9%	4.0%	10.4%	22.9%^a^
Hypertension	28.7%	29.6%^b^	28.3%	29.7%	7.4%	28.0%	55.0%^a^
Number of metastasis sites at index, mean (SD)	1.67 (0.94)	1.57 (0.82)^b^	1.85 (0.95)	1.48 (0.81)^a^	1.55 (0.83)	1.65 (0.91)	1.51 (0.79)^b^
Site of metastasis at index							
Liver	20.2%	14.4%^a^	17.9%	16.8%	14.1%	18.0%	17.0%
Lung	16.4%	10.9%^a^	9.8%	15.7%^a^	12.1%	13.4%	15.3%
Bone	29.8%	42.2%^a^	35.7%	36.5%	36.6%	35.6%	38.2%
Brain	16.8%	11.4%^a^	8.0%	17.6%^a^	12.9%	14.2%	14.4%

*N* = number of patients meeting study selection criteria.

^a^ *P* value compared to corresponding HER2-targeted agent subgroup(s) <0.0001.

^b^ *P* value compared to corresponding HER2-targeted agent subgroup(s) <0.01.

^c^ *P* value compared to corresponding HER2-targeted agent subgroup(s) <0.05.

^d^EPO: exclusive provider organizations, PPO: preferred provider organization plans, POS: point-of-service, HMO: health maintenance organization, CDHP: consumer-driven health plan, and HDHP: high deductible health plan.

^e^The denominator for the percentages is the number of patients with a diagnosis of an earlier stage breast cancer.

^f^Percent of patients with surgery in the preindex period.

**Table 3:** Healthcare utilization during follow-up period.

	No Treatment	No HER2-targeted agents		HER2-targeted agents	
	*N* = 2,590	*N* = 12,840		*N* = 2,629	
	Mean (SD)	Mean (SD)		Mean (SD)	
*All-cause monthly utilization*(*PPPM*)^e^					
Inpatient admissions	0.29 (1.17)	0.15 (0.54)		0.14 (0.65)^a^	
Total inpatient days	1.65 (4.87)	0.87 (3.03)		0.74 (2.32)^a^	
Outpatient utilization	11.49 (14.83)	14.10 (12.93)		16.72 (11.36)^a^	
Emergency department (ED) visits	0.17 (0.61)	0.12 (0.42)		0.13 (0.51)^a^	
Outpatient office visits	1.30 (2.25)	1.70 (1.23)		1.94 (1.17)^a^	
Radiation treatment^c^	2.22 (7.60)	2.75 (6.58)		3.05 (5.57)^a^	
Diagnostic radiology^c^	1.73 (3.31)	1.78 (2.47)		2.17 (2.39)^a^	
Laboratory services	2.96 (5.69)	4.32 (5.45)		5.27 (5.06)^a^	
Other outpatient care^d^	3.11 (4.32)	3.42 (3.45)		4.17 (3.11)^a^	
Prescription fills	2.42 (2.65)	3.23 (2.50)		3.16 (2.42)^a^	
*Breast cancer monthly utilization*(*PPPM*)^c^					
Inpatient admissions	0.07 (0.40)	0.01 (0.17)		0.01 (0.06)^a^	
Total inpatient days	0.47 (3.03)	0.07 (0.63)		0.08 (0.70)^a^	
Outpatient utilization	5.01 (10.04)	8.09 (9.71)		11.00 (8.84)^a^	
Emergency department (ED) visits	0.03 (0.30)	0.02 (0.17)		0.03 (0.18)^b^	
Outpatient office visits	0.65 (2.00)	1.04 (1.01)		1.36 (1.02)^a^	
Radiation treatment^c^	1.12 (5.58)	1.63 (5.07)		1.81 (4.21)^a^	
Diagnostic radiology^c^	0.68 (2.21)	0.87 (1.55)		1.30 (1.73)^a^	
Laboratory services	1.31 (3.84)	2.79 (4.38)		3.92 (4.13)^a^	
Other outpatient care^d^	1.20 (2.42)	1.72 (2.03)		2.55 (2.27)^a^	
Office-administered antineoplastic agents	n/a	0.01 (0.07)		0.04 (0.15)^a^	
Outpatient pharmacy antineoplastic agents	n/a	0.28 (0.34)		0.27 (0.35)	

^a^ *P* value compared with no treatment and no HER2-targeted agents <0.0001.

^b^ *P* value compared with no treatment and no HER2-targeted agents <0.05.

^c^Radiation treatment and diagnostic radiology encompass all outpatient radiology services during follow-up.

^d^Other outpatient care includes all remaining outpatient services that are not reported individually. ^e^Patient monthly utilization is calculated using the following formula: (patient's total number visits or claims/patient's total days of follow-up) ∗ 30 days.

**Table 4:** Healthcare utilization during follow-up period among HER2-targeted agent subgroups.

	HT−	HT+	*De novo *	Recurrent	AGE 18–44	AGE 45–64	AGE 65+
	*N* = 1,272	*N* = 1,357	*N* = 975	*N* = 1,654	*N* = 404	*N* = 1,801	*N* = 424
	Mean (SD)	Mean (SD)	Mean (SD)	Mean (SD)	Mean (SD)	Mean (SD)	Mean (SD)
*All-cause monthly utilization*(*PPPM*)^f^							
Inpatient admissions	0.20 (0.90)	0.09 (0.23)^a^	0.11 (0.38)	0.16 (0.77)^c^	0.10 (0.25)	0.16 (0.76)	0.12 (0.33)
Total inpatient days	1.00 (2.92)	0.50 (1.53)^a^	0.62 (2.08)	0.81 (2.45)^c^	0.52 (1.64)	0.80 (2.52)	0.69 (1.98)
Outpatient utilization	17.95 (12.33)	15.57 (10.23)^a^	17.71 (11.19)	16.14 (11.42)^b^	17.53 (11.47)	17.11 (11.06)	14.28 (12.16)^a^
Emergency department (ED) visits	0.17 (0.70)	0.08 (0.21)^a^	0.09 (0.26)	0.14 (0.61)^b^	0.11 (0.32)	0.12 (0.57)	0.15 (0.38)
Outpatient office visits	2.03 (1.27)	1.85 (1.07)^a^	2.05 (1.15)	1.87 (1.18)^a^	2.05 (1.38)	1.92 (1.14)	1.90 (1.10)
Radiation treatment^d^	3.25 (6.08)	2.86 (5.04)	3.17 (4.54)	2.98 (6.10)	3.15 (5.15)	3.10 (5.62)	2.75 (5.76)
Diagnostic radiology^d^	2.34 (2.78)	2.01 (1.95)^b^	2.35 (2.72)	2.06 (2.17)^b^	2.25 (2.02)	2.20 (2.41)	1.96 (2.61)
Laboratory services	5.62 (5.57)	4.94 (4.50)^b^	5.71 (5.26)	5.01 (4.91)^b^	6.19 (5.32)	5.61 (4.95)	2.95 (4.57)^a^
Other outpatient care^e^	4.53 (3.58)	3.82 (2.54)^a^	4.33 (3.26)	4.07 (3.01)^c^	3.77 (2.16)	4.16 (2.91)	4.57 (4.39)^b^
Prescription fills	3.08 (2.51)	3.25 (2.32)	3.14 (2.33)	3.18 (2.47)	2.83 (2.35)	3.17 (2.39)	3.46 (2.54)^b^
*Breast cancer monthly utilization*(*PPPM*)^c^							
Inpatient admissions	0.02 (0.08)	0.01 (0.03)^b^	0.02 (0.06)	0.01 (0.06)^a^	0.01 (0.05)	0.01 (0.06)	0.01 (0.04)
Total inpatient days	0.12 (0.98)	0.04 (0.20)^b^	0.10 (0.91)	0.06 (0.53)	0.04 (0.24)	0.09 (0.80)	0.04 (0.47)
Outpatient utilization	11.80 (9.52)	10.24 (8.07)^a^	12.58 (8.96)	10.07 (8.63)^a^	12.28 (9.88)	11.35 (8.30)	8.31 (9.46)^a^
Emergency department (ED) visits	0.04 (0.20)	0.02 (0.15)^c^	0.02 (0.15)	0.03 (0.19)	0.04 (0.21)	0.02 (0.16)	0.03 (0.19)
Outpatient office visits	1.47 (1.12)	1.25 (0.90)^a^	1.51 (1.02)	1.27 (1.00)^a^	1.48 (1.27)	1.36 (0.97)	1.24 (0.90)^b^
Radiation treatment^d^	1.90 (4.66)	1.72 (3.74)	2.24 (3.64)	1.55 (4.50)^a^	2.08 (4.55)	1.84 (4.16)	1.43 (4.08)
Diagnostic radiology^d^	1.39 (1.83)	1.21 (1.63)^b^	1.55 (1.91)	1.15 (1.60)^a^	1.48 (1.73)	1.33 (1.72)	0.98 (1.75)^a^
Laboratory services	4.15 (4.40)	3.71 (3.84)^b^	4.39 (4.41)	3.65 (3.92)^a^	4.71 (4.38)	4.16 (3.98)	2.18 (4.04)^a^
Other outpatient care^e^	2.81 (2.71)	2.30 (1.72)^a^	2.82 (2.66)	2.38 (1.98)^a^	2.46 (1.65)	2.60 (2.07)	2.42 (3.32)
Office-administered antineoplastic agents	0.04 (0.16)	0.05 (0.13)	0.05 (0.15)	0.04 (0.15)	0.04 (0.14)	0.05 (0.15)	0.04 (0.14)
Outpatient pharmacy antineoplastic agents	0.12 (0.25)	0.41 (0.37)^b^	0.25 (0.29)	0.28 (0.38)^c^	0.33 (0.36)	0.27 (0.35)	0.24 (0.34)^b^

^a^ *P* value compared to corresponding HER2-targeted agent subgroup(s) <0.0001.

^b^ *P* value compared to corresponding HER2-targeted agent subgroup(s) <0.01.

^c^ *P* value compared to corresponding HER2-targeted agent subgroup(s) <0.05.

^d^Radiation treatment and diagnostic radiology encompass all outpatient radiology services during follow-up.

^e^Other outpatient care includes all remaining outpatient services that are not reported individually.

^f^Patient's monthly utilization is calculated using the following formula: (patient's total number visits or claims/patient's total days of follow-up) ∗ 30 days.

**Table 5:** Unadjusted healthcare costs during follow-up.

	No treatment	No HER2-targeted agents	HER2-targeted agents
	*N* = 2,590	*N* = 12,840	*N* = 2,629
	Mean (SD)	Mean (SD)	Mean (SD)
*All-cause monthly expenditures*(*PPPM*)^c^			
Inpatient	$6,683 (27,528)	$3,416 (13,754)	$3,327 (14,132)^a^
Outpatient visits and services	$4,524 (7,794)	$5,910 (7,031)	$9,939 (8,453)^a^
Emergency department (ED) visits	$96 (426)	$95 (657)	$119 (1,150)
Outpatient office visits	$220 (703)	$220 (335)	$254 (387)^b^
Radiation treatment^d^	$427 (1,815)	$588 (1,805)	$734 (1,646)^a^
Diagnostic radiology^d^	$1,414 (4,073)	$1,083 (2,592)	$1,462 (2,630)^a^
Laboratory services	$309 (1,136)	$263 (660)	$287 (567)^b^
Other outpatient care^e^	$2,058 (4,397)	$3,661 (5,024)	$7,083 (6,640)^a^
Prescription fills	$286 (681)	$502 (736)	$839 (1,298)^a^
Total healthcare	**$11,493 (29,357)**	**$9,828 (16,182)**	**$14,105 (16,865)** ^ a^
*Breast cancer monthly expenditures*(*PPPM*)^c^			
Inpatient	$1,525 (13,107)	$271 (2,519)	$338 (3,139)^a^
Outpatient visits and services	$2,567 (5,952)	$4,021 (5,758)	$7,858 (7,603^a^)
Emergency department (ED) visits	$25 (216)	$35 (471)	$58 (1,054)
Outpatient office visits	$134 (642)	$142 (285)	$185 (343)^a^
Radiation treatment^d^	$221 (1,295)	$387 (1,513)	$472 (1,376)^a^
Diagnostic radiology^d^	$1,018 (3,502)	$776 (2,225)	$1,114 (2,315)^a^
Laboratory services	$267 (1,421)	$208 (552)	$253 (616)^b^
Other outpatient care^e^	$904 (2,907)	$2,106 (3,657)	$4,177 (4,627)^a^
Office-administered antineoplastic agents	$0 (0)	$367 (1,377)	$1,598 (3,418)^a^
Outpatient pharmacy antineoplastic agents	$0 (0)	$89 (225)	$390 (867)^a^
Total healthcare	**$4,092 (14,548)**	**$4,382 (6,313)**	**$8,585 (8,285)** ^ a^

^a^ *P* value compared with no treatment and no HER2-targeted agents <0.0001.

^b^ *P* value compared with no treatment and no HER2-targeted agents <0.01.

^c^Patient monthly expenditures were calculated using the following formula: (patient total expenditures/patient total days of follow-up) ∗ 30 days.

^d^Radiation treatment and diagnostic radiology encompass all outpatient radiology services during follow-up.

^e^Other outpatient care includes all remaining outpatient services that are not reported individually.

**Table 6:** Unadjusted healthcare costs during follow-up period among HER2-targeted agent users.

	HT−	HT+	*De novo *	Recurrent	AGE 18–44	AGE 45–64	AGE 65+
	*N* = 1,272	*N* = 1,357	*N* = 975	*N* = 1,654	*N* = 404	*N* = 1,801	*N* = 424
	Mean (SD)	Mean (SD)	Mean (SD)	Mean (SD)	Mean (SD)	Mean (SD)	Mean (SD)
*All-cause monthly expenditures*(*PPPM*)^f^							
Inpatient admissions	$4,623 (18,646)	$2,111 (7,605)^a^	$3,129 (13,402)	$3,443 (14,548)	$2,249 (5,461)	$3,914 (16,706)	$1,861 (4,470)^b^
Outpatient visits and services	$10,493 (8,752)	$9,422 (8,130)^b^	$11,517 (8,661)	$9,009 (8,190)^a^	$11,166 (8,494)	$10,334 (8,497)	$7,091 (7,597)^a^
Emergency department (ED) visits	$138 (766)	$101 (1,418)	$101 (618)	$129 (1,370)	$129 (744)	$118 (1,277)	$113 (869)
Outpatient office visits	$270 (434)	$240 (335)	$287 (491)	$235 (308)^b^	$260 (214)	$264 (434)	$207 (281)^b^
Radiation treatment^d^	$817 (1,945)	$655 (1,300)^c^	$765 (1,310)	$715 (1,815)	$870 (1,527)	$751 (1,532)	$531 (2,136)^b^
Diagnostic radiology^d^	$1,501 (2,521)	$1,425 (2,728)	$1,436 (2,486)	$1,477 (2,712)	$1,647 (2,607)	$1,544 (2,699)	$939 (2,271)^a^
Laboratory services	$311 (657)	$265 (464)^c^	$301 (446)	$279 (627)	$349 (562)	$305 (612)	$153 (277)^a^
Other outpatient care^e^	$7,456 (6,965)	$6,737 (6,301)^b^	$8,626 (7,190)	$6,174 (6,116)^a^	$7,911 (6,727)	$7,353 (6,703)	$5,148 (5,907)^a^
Prescription fills	$818 (1,369)	$858 (1,227)	$577 (845)	$994 (1,481)^a^	$923 (1,355)	$848 (1,309)	$722 (1,185)
Total healthcare	**$15,934 (20,928)**	**$12,391 (11,591)** ^ a^	**$15,223 (16,448)**	**$13,446 (17,078)** ^ b^	**$14,338 (10,828)**	**$15,096 (19,091)**	**$9,674 (8,982)** ^ a^
*Breast cancer monthly expenditures*(*PPPM*)^f^							
Inpatient	$476 (4,374)	$207 (1,054)^c^	$601 (4,758)	$183 (1,503)^b^	$246 (1,181)	$400 (3,654)	$159 (1,737)
Outpatient visits and services	$8,258 (7,793)	$7,475 (7,401)^b^	$9,582 (7,961)	$6,841 (7,195)^a^	$8,916 (7,912)	$8,201 (7,573)	$5,388 (6,911)^a^
Emergency department (ED) visits	$50 (431)	$65 (1,407)	$43 (427)	$67 (1,288)	$52 (403)	$64 (1,247)	$36 (363)
Outpatient office visits	$200 (369)	$171 (317)^c^	$216 (418)	$167 (289)^b^	$190 (195)	$194 (382)	$144 (269)^c^
Radiation treatment^d^	$524 (1,682)	$423 (1,006)	$568 (1,115)	$416 (1,507)^b^	$591 (1,295)	$484 (1,245)	$309 (1,879)^c^
Diagnostic radiology^d^	$1,133 (2,136)	$1,095 (2,470)	$1,167 (2,218)	$1,083 (2,370)	$1,282 (2,484)	$1,182 (2,356)	$668 (1,880)^a^
Laboratory services	$264 (640)	$243 (592)	$281 (578)	$236 (637)	$317 (793)	$264 (593)	$145 (493)^b^
Other outpatient care^e^	$4,497 (5,260)	$3,873 (3,919)^b^	$5,155 (5,200)	$3,601 (4,418)^a^	$4,690 (4,867)	$4,342 (4,562)	$2,989 (4,483)^a^
Office-administered antineoplastic agents	$1,590 (3,186)	$1,606 (3,620)	$2,152 (3,555)	$1,272 (3,292)^a^	$1,794 (3,401)	$1,672 (3,619)	$1,098 (2,346)^ c^
Outpatient pharmacy antineoplastic agents	$369 (890)	$410 (844)	$227 (534)	$487 (1,001)^a^	$426 (798)	$403 (894)	$303 (804)
Total healthcare	**$9,103 (9,000)**	**$8,092 (7,521)** ^ b^	**$10,409 (9,334)**	**$7,510 (7,394)** ^ a^	**$9,589 (8,103)**	**$9,004 (8,472)**	**$5,850 (7,020)** ^ a^

^a^ *P* value compared to corresponding HER2-targeted agent subgroup(s) <0.0001.

^b^ *P* value compared to corresponding HER2-targeted agent subgroup(s) <0.01.

^c^ *P* value compared to corresponding HER2-targeted agent subgroup(s) <0.05.

^d^Radiation treatment and diagnostic radiology encompass all outpatient radiology services during follow-up.

^e^Other outpatient care includes all remaining outpatient services that are not reported individually.

^f^Patient's monthly expenditures were calculated using the following formula: (patient's total expenditures/patient's total days of follow-up) ∗ 30 days.

**Table 7:** Adjusted expenditures.

	*N*	All-cause healthcare expenditures			Breast cancer-related expenditures		
		Mean	Lower 95% CI	Upper 95% CI	Mean	Lower 95% CI	Upper 95% CI
*HER2-targeted agent status *							
HER2-targeted agents	18,059	$11,107	$10,376	$11,838	$6,215	$5,734	$6,696
No HER2-targeted agents	18,059	$8,458	$8,266	$8,651	$3,908	$3,816	$4,000
No treatment	18,059	$8,284	$7,925	$8,642	$2,858	$2,713	$3,003
Incremental Expenditures HER2-targeted agents versus no HER2-targeted agents		$2,649	$1,892	$3,405	$2,307	$1,817	$2,796
Incremental Expenditures HER2-targeted agents versus no treatment		$2,824	$2,009	$3,638	$3,357	$2,855	$3,859
*HER2-targeted agent patients *							
HT status							
HT+	2,629	$10,359	$9,849	$10,869	$6,869	$6,487	$7,251
HT−	2,629	$12,924	$12,266	$13,582	$7,757	$7,317	$8,198
Incremental expenditures		($2,565)	($3,397)	($1,733)	($888)	($1,472)	($305)
*De novo* versus recurrent							
*De novo *	2,629	$12,139	$11,438	$12,840	$8,200	$7,674	$8,726
Recurrent	2,629	$11,184	$10,689	$11,679	$6,791	$6,450	$7,131
Incremental expenditures		$955	$97	$1,813	$1,409	$782	$2,036
Age group							
Age 18–44	2,629	$13,650	$12,670	$14,630	$9,075	$8,345	$9,806
Age 45–64	2,629	$13,101	$12,655	$13,547	$8,193	$7,878	$8,509
Age 65+	2,629	$8,570	$7,916	$9,224	$5,198	$4,744	$5,652
Incremental expenditures Age 18–44 versus age 45–64		$549	($528)	$1,626	$882	$87	$1,678
Incremental expenditures Age 65+ versus age 45–64		($4,531)	($5,323)	($3,739)	($2,995)	($3,548)	($2,443)
